# Supplementary material for: A time-resolved meta-analysis of consensus gene expression profiles during human T-cell activation
Source: Genome Biol. 2023 Dec 14;24:287. doi: 10.1186/s13059-023-03120-7 (PMC10722659; doi:10.1186/s13059-023-03120-7)
Supplement: Supplementary file 2 — Additional file 2. Overview of all genes of the consensus signatures and their temporal expression as an interactive HTML document. In addition, the signaling pathways of the enrichment analyses for the consensus signatures are shown. [file 13059_2023_3120_MOESM2_ESM.html]

Additional file 2


# Additional file 2

### A time-resolved meta-analysis of consensus gene expression profiles during human T-cell activation

Author: Michael Rade


# 1 Consensus gene expression profiles

Fig. 4C of the publication: Consensus gene expression profiles for CD4+ T-cells from the Discovery Set. We grouped the consensus expression profiles over the course by genes associated with identity and shared metagenes. Each boxplot represents one CD4+ T-cell population from the Discovery Set. The y-axis depicts standardized median expression of genes from samples with identical analysis time points. The number in parentheses represents the number of genes for the corresponding metagene

## 1.1 Summary

Shown are all genes from the consensus signatures that we identified based on the *Discovery Set*.

- **Mg:** Assignment of genes to metagenes based on the *Discovery Set*
- **Time\_point:** Time point of activation (hours) with the highest absolute “confect” value from the meta-analysis.
- **Effect:** Combined effect size from the meta-analysis for the time point with the highest absolute “confect” value.
- **P\_value:** Adjusted p-values for the combined effect size using the method of Benjamini-Hochberg.
- **Confect:** Confident effect sizes or “confect” for the combined effect size from the meta-analysis.
- **Passed\_V1:** Boolean value indicating whether the genes have passed the *Memory T-cell Verification set*.
- **Passed\_V2:** Boolean value indicating whether the genes have passed the *Pan T-cell Verification set*.
- **Mg\_V1/V2:** Assignment of genes to metagenes in the 2 *Verification Sets*. The following special cases are possible:
  - Due to the lack of analysis time points (0.5 to 4 hours) in the *Pan T-cell Verification set* (V2), we annotated metagene M2 in column “Mg\_V2” as “unknown”.
  - n.s = A gene was not significant (FDR <0.05) in any contrast of the differential gene expression analysis.
  - n.p = A gene was not available in den datasset (Only occurs in V1).
- **Passed\_V2\_Neg\_Ctrl:** Filtering step for time series negative controls (unactivated Pan T-cells after 6 to 72 hours) when comparing their gene expression profiles with the kinetics of the activated Pan T-cells. A boolean of “True” means that the gene passed the filtering step.
- **Pass\_Housekeeping\_Filter:** Genes that are present in the “Housekeeping and Reference Transcript Atlas database” (PMID: 32663312). A boolean of “True” means that the gene is not present in the database.

## 1.2 Gene expression profiles

Shown are all genes from the consensus signatures that we identified based on the *Discovery Set*. If you click on the gene and press the “Backspace” key, you can then search for a gene by typing the symbol.

- **D:** Discovery set
- **V1: Th0 (Memory):** Memory T-cell Verification Set\*\*
- **V2: Th0 (Pan):** Pan T-cell Verification set\*\*.
- **V2: Neg. ctrl. (Pan):** Pan T-cell Verification set (negative controls)\*\*.

Search for a gene

The y-axis represents the median expression of genes from samples with identical analysis time points. The x-axis depicted quantile normalized count-per-million (CPM) for RNA-Seq and the normalized intensities (RMA normalization) for microarrays (Th1, Th2). Vertical lines represent the interquartile ranges.

# 2 Enrichment analysis

- Enrichment analysis test settings for the R/Bioconductor package clusterProfiler
  - P-value cutoff: 0.05 (cutoff for adjusted p-values)
  - Correction for multiple testing: Benjamini-Hochberg
  - Minimal size of genes annotated by term for testing: 10
  - Maximal size of genes annotated by term for testing: 500
  - Background gene set: All GENCODEv29 genes

- Meaning of BgRatio (M/N) and GeneRatio (k/n)
  - M = size of the gene-set (e.g. number of genes in the cell cycle term). More precisely, the total number of genes (ENTREZ Gene IDs) from the universe found in this gene-set.
  - N = size of all unique genes in the collection of gene-sets (e.g. the GO term collection for BPs). More precisely, the total number of genes (ENTREZ Gene IDs) from the universe found in this collection
  - k = size of overlap of genes with a specific gene-set (e.g. cell cycle term). Only unique ENTREZ Gene IDs were considered.
  - n = size of overlap of genes with all genes in the collection of gene-sets (e.g. the GO term collection for BPs). Only unique ENTREZ Gene IDs were considered.
  - The rich factor is k / M

## 2.1 Discovery Set

All genes from the consensus signatures that we identified based on the *Discovery Set* were used for enrichment analysis.

### 2.1.1 Gene Ontology term enrichment

Gene ontology category: Biological Process

### 2.1.2 Reactome pathway enrichment

## 2.2 Verified by the Memory T-cell Verification Set

All genes from the consensus signatures that we identified based on the *Discovery Set* and verified for temporal consistency by the *Memory T-cell Verification Set* were used for enrichment analysis.

### 2.2.1 Gene Ontology term enrichment

Gene ontology category: Biological Process

### 2.2.2 Reactome pathway enrichment

## 2.3 Verified by both Verificaton Sets

All genes from the consensus signatures that we identified based on the *Discovery Set* and verified for temporal consistency by the *Memory* and *Pan T-cell Verification Set* were used for enrichment analysis.

### 2.3.1 Gene Ontology term enrichment

Gene ontology category: Biological Process

### 2.3.2 Reactome pathway enrichment
