# Supplementary material for: A time-resolved meta-analysis of consensus gene expression profiles during human T-cell activation
Source: Genome Biol. 2023 Dec 14;24:287. doi: 10.1186/s13059-023-03120-7 (PMC10722659; doi:10.1186/s13059-023-03120-7)
Supplement: Supplementary file 4 — Additional file 4. Peer review history. [file 13059_2023_3120_MOESM4_ESM.docx]

**Review history**

**First round of review**

**Reviewer 1**

General comments
The manuscript by Rade and colleagues describes a computational-experimental study addressing gene expression profiles of human T cells during activation. The authors analyzed publicly available time-series data and identified gene expression profiles for different time points, and verified the consensus signature using another published dataset as well as a dataset generated using T cells from healthy donors. Further, a sc-RNA seq data set from published CAR T-cells study was used and analyzed with respect to the signatures.
Given the impact of cancer immunotherapy in general, and CAR-T cells therapy specifically, comprehensive characterization of the gene expression profiles of human T cells during activation is highly welcome and could be of interest to a broader audience. The authors addressed this challenge using up-to-date available data and sound methods. However, the study is mostly descriptive and there are no novel insights. Of note, albeit also descriptive studies can be a valuable resource, there are several concerns that need to be addressed. First and most importantly, there is no clear distinction throughout the study between different T cell subsets. The bulk of the data used is from CD4 T cells whereas the validation data is from CD4 and CD8 T cells. Thus, it is not clear what is the contribution of the CD8 subset to the results. Moreover, the pan T-cell verification used unspecific ex-vivo stimulation (anti-CD3/CD28 beads). How does the signaling differ between unspecific and antigen-specific stimulation in CD4 and CD8 subsets and between ex-vivo and in-vivo stimulation? Further, CAR-T cell activation and intracellular signaling differs from the native TCR-signaling due to the truncated receptor and it is unclear how relevant is the analysis. Finally, there are several methodological issues that need to be addressed (see specific comments).


Specific comments
1. The authors used RNA-seq data and microarray data, which have different dynamic ranges, and hence, the integration of both datasets is not straightforward. How was this issue solved?
2. Non-negative matrix factorization is inherently not exactly solvable. In order to demonstrate that the results are robust, an additional techniques should be applied.
3. The data reported in Figure 6C is significant but not very strong given the large overlap of the expression values. The reporting and the discussion should be toned down.
4. The Methods and the Results section are not clearly separated since some methodological parts are included in the Results section. The manuscript should be rewritten to distinguish the two.
5. The discussion on the various putative genes should shortened since the genes have not been validated using orthogonal methods.

**Reviewer 2**

In this study, Rade et al. present a first comprehensive analysis of temporal consensus gene expression signatures across T-cell populations. The authors used non-negative matrix factorization (NMF) for their analysis. They first analyzed publicly available datasets as a discovery set and then verified their findings by performing RNA-seq. Consequently, they defined temporally resolved gene expression patterns across T-cell populations after TCT stimulation, resulting in identification of 3-5 metagenes. They also used scRNA-seq datasets from large B-cell lymphoma (LBCL) patients and examined whether expression of the metagenes they identified were correlated with immune effector cell-associated neurotoxicity syndrome (ICNAS) grades.
While the findings from this study are interesting and could improve our understanding of T-cell activation after TCR stimulation, further evidence and/or analysis that support their conclusion seem to be needed. Several specific comments are as follows:
1. Biological significance of profiling the "consensus" gene expression across different T-cell populations seems to be unclear.
(1) If the authors could address molecular mechanisms underlying how memory T-cells respond to antigenic stimulation faster than naïve T-cells, the present manuscript would be improved and of interest to many immunologists. Are M2-M5 induced faster in Memory T-cells compared to naïve T-cells?
(2) Why don't the authors show any result from the Memory T-cell Verification Set in main figures? Alternatively, they can sort memory T-cells from some healthy donors and perform RNA-seq to address the point raised in (1).

2. Relationship between Figure 2A and B is unclear.
(1) For examples, among 5 populations, the authors identified 3641 DEGs after 24 hours of activation as described on line 337. They also identified about 3000 DEGs at each time point according to Figure 2A. Based on Figure 2A, they identified approximately 3000+3641+3500+3000 = 13141 DEGs if there are no overlap. As shown in Figure 2B, they identified 4808+26+55+67+14+7920 = 12890 DEGs. How do these two numbers (13141 and 12890) link? In addition, on lines 673-674, the authors argue that 7920 genes were upregulated in all 5 population "at each contrast" (e.g. 12h vs. 0h). The meaning of "at each contrast" is unclear for this reviewer.
(2) For another example, as show in black in Figure 2A, they identified >10000 DEGs at each time point except at 1h, resulting in identification of at least 70000 DEGs. Based on Figure 2B, they identified 17867+14097= 31964 DEGs. How do these two numbers (i.e. 70000 and 31964) link? Is there any overlap?
(3) This reviewer assumes that union of DEGs at each time point is equal to summation of gene number in each color. Venn diagram would be helpful for understanding the relationship between Figure 2A and B.

Minor points
1. In Figure 6B, "standardized average expression" should be added beside the color key.
2. In Figure 6C, it is supposed that T-cells are more active in patients with higher grade of ICANS than those with lower grade. However, the authors observed an opposite result: T-cells showed decreased expression of M5, M3, and M3/M5 in patients with higher grade of ICANS. The authors should discuss this matter.
3. On line 542, a term "early-response metagene (M1)" seems to be strange because expression of M1 decreases rapidly after TCR stimulation. "early-response" is more suitable for M2, which increases rapidly after TCR stimulation. Please consider renaming of M1.

**Authors’ response to reviewers**

# Reviewer #1:

## General comments

The manuscript by Rade and colleagues describes a computational-experimental study addressing gene expression profiles of human T cells during activation. The authors analyzed publicly available time- series data and identified gene expression profiles for different time points, and verified the consensus signature using another published dataset as well as a dataset generated using T cells from healthy donors. Further, a sc-RNA seq data set from published CAR T-cells study was used and analyzed with respect to the signatures.

Given the impact of cancer immunotherapy in general, and CAR-T cells therapy specifically, comprehensive characterization of the gene expression profiles of human T cells during activation is highly welcome and could be of interest to a broader audience. The authors addressed this challenge using up-to-date available data and sound methods. However, the study is mostly descriptive and there are no novel insights. Of note, albeit also descriptive studies can be a valuable resource, there are several concerns that need to be addressed.

**Comment 1:** First and most importantly, there is no clear distinction throughout the study between different T cell subsets.

**Answer:** Thank you very much for this important advice. We agree with the reviewer that describing gene expression changes during T cell activation that are specific to a T-cell population would be of high value. It is indeed not easy to provide distinct longitudinal gene expression signatures between different T cell subsets. For robust signatures for each T cell subset, an adequate number of replicates for each time point and T cell fate is needed, but the currently available data sets, unfortunately, do not fulfill this requirement. The Th1, Th2 and Th17 populations in the *Discovery Dataset* consist of only three biological replicates, which is not sufficient to guarantee robustness in independent samples (see Figure 1 below).

Characterizing a consensus gene expression profile for human T cell activation is of great value to reveal characteristics of an overall immune response no matter of the stressor and immune condition. Our rationale is that the distribution of Th1, Th2, Th17 and Th0 in human peripheral blood differs greatly from immune conditions to immune conditions (e.g. cytokine storm or allergy). Although there may be clear tendencies towards certain subpopulations for well-described inflammatory diseases, for example under allergy to Th2, even these distributions vary greatly between individuals. Therefore, we determined a consensus gene expression signature that identifies T cells upon their activation across lineage choices, independently from the underlying inflammatory disease condition (e.g. inflammation vs. hypersensitivity reaction). The signature covers T cell activation independent of cellular heterogeneity.

In future, it would be great to expand the signature towards fate choices that underpin the nature of the inflammatory responses. However, defining longitudinal gene expression signatures for the different fate choices requires a sufficient number of replicates for each time point and T cell fate. Unfortunately, the currently available data sets do not meet this requirement. Because of this limitation, we are currently not able to expand the consensus signature to robust expression profiles for individual T cell fates.

**Adjustments in the manuscript:** To provide a first glance of distinct gene sets between different T-cell population, we now provide an additional figure Additional file 1: Figure S8 (Figure 2 below) that gives an overview of the number of genes that are significantly up-regulated (or down-regulated) during one T-cell fate and significantly down-regulated (or up-regulated) for all other T-cell fates.

**Figure 1:** from Additional file 1: Supplemental Figure S1.

**Figure 2 (new Supplemental Figure S8): Scatter plot of significantly differentially expressed (DE) genes for each T-cell population showing opposite log fold change (logFC) compared to all other populations.** For each CD4+ T-cell population in the Discovery Set and at each time point of activation, we assessed differential gene expression between polarized or activated T-cells without differentiating cytokines and CD4+ T-cells before activation. We declared a gene as DE if the FDR- adjusted p-value was <0.05. Only genes that are significant in all populations at a given activation time point and have the same logFC direction in all but one population are shown. For Th2, due to a high number of DE genes, we annotated only

the top three ranked genes (by logFC) for each time point (if available).

**Comment 2:** The bulk of the data used is from CD4 T cells whereas the validation data is from CD4 and CD8 T cells. Thus, it is not clear what is the contribution of the CD8 subset to the results.

**Answer:** Thank you very much for pointing this out. We designed the cell composition of the Verification Set to be relevant for human peripheral blood in various immune conditions. We used the Pan T cell Verification Set to exclude from the consensus signature those genes whose expression profiles deviated from conditions as close as possible to human blood (i.e. conditions where cells are not sorted prior to analysis). We could not include such a set during training, i.e. the Discovery Set, because no gene expression time series for Pan T cells were published at that time. In fact, we see our study as the very first one describing Pan T cell activation as time series. The cell composition of the Pan T cell Verification Set comprised in majority CD4+ T cells and only a certain proportion of CD8+ T cells (see Figure 3 below which is a copy of Figure 5B in the manuscript). Thus, we expect the influence of the CD8+ cells to be minor, but relevant for applying the consensus gene signature in conditions close to human immune biology. For example, this enables us to check applicability of the consensus T cell signature in a case study by evaluating the activation signature from infusion bags containing a mixture of unspecific stimulated CD4 and CD8-bearing CD19 CAR T cells.

**Adjustments in the manuscript:** To make this point clearer in the manuscript, we adapted the description of rationale of the Pan T cell verification study in Section “4.5 Verification and mapping to a wider variety of T-cell transcriptional landscape”. It now reads: “We used this dataset as *Pan T-cell Verification Set* to exclude from the consensus signature those genes whose expression profiles

deviated from conditions as close as possible to human blood (i.e. conditions where cells are not sorted prior to analysis). The fractions of the Pan T-cell populations are shown in Fig. 5B.”

**Figure 3:** Copied from Figure 5B from the manuscript. Depicted are the fractions of Pan T-cell populations before activation.

200.000 cells were analyzed using seven human blood donors (see Additional file 1 for details).

**Comment 3:** Moreover, the pan T-cell verification used unspecific ex-vivo stimulation (anti-CD3/CD28 beads). How does the signaling differ between unspecific and antigen-specific stimulation in CD4 and CD8 subsets and between ex-vivo and in-vivo stimulation?

**Answer:** We thank the reviewer for this careful advice. We agree with the reviewer that it is of great interest to characterize T cell activation upon antigen-specific stimulation, preferably under in vivo conditions. We are aware that the consensus signatures developed, are based on an artificial immune response where T-cells were activated with anti-CD3/anti-CD28 coated beads and that such an experimental design only partially reflects human immune response in-vivo. Therefore, the expression pattern of the genes from our signatures might behave differently under in-vivo conditions. To account for that we carefully selected CAR T cells infusion bags, i.e. (CAR) T cells cultured and expanded with anti-CD3/anti-CD28 beads during manufacturing, as a suitable use case.

Further, we assume that central signal transduction pathways are similar upon nonspecific and antigen- specific stimulation as shown by multiple studies [1–10]. For example, cell cycle entry, cell growth, IL2 signaling, metabolism driving T cell activation, and mitochondrial biogenesis are part of the hallmarks of T cell activation [11]. We compared the results of nonspecific activation with these hallmarks and observed a significant enrichment (see Figures 4E, S12-14, S18 and S19 in the manuscript).

However, since it is of great interest to characterize T cell activation upon antigen-specific stimulation, we are already investigating the temporal resolution of the recall immune response to antigens in an ongoing study. The experimental design is outlined in Figure 4A below. It should be noted that a recall immune response is actually highly heterogeneous between donors. Therefore, a high number of replicates is required to achieve sufficient statistical power. Figure 4 (B and C) depicts the relation of log fold changes and variance for the first four donor samples. Unfortunately, n=4 replicates are much too low to obtain robust results due to high inter-individual variability reflected by low confect values (see Figure 4B and 4C below). Hence, we refrain to report the first results of the ongoing study in this manuscript. We are continuing our work to increase sample size and intend to publish the new results separately. In fact, to our knowledge, this will be the very first study describing T cell activation upon antigen-specific stimulation as time series.

**Figure 4: (A)** We isolated Pan T-cells from 4 healthy donors. RNA-Seq was performed at 4 different time points of anti- CD3/anti-CD28 activation and tetanus toxin (12, 24, 48 and 72 hours) and from unactivated (0 hours) Pan T-cells. We additionally cultured and sequenced Pan T-cells as negative controls in serum-free medium at the same time points as mentioned above. We performed DGEA to find differentially expressed genes for activated (beads and tetanus toxin) and unactivated T-cells (medium) at 12 to 72 hours compared to unactivated T-cells at 0 hours. Red dots represent DE genes (FDR <0.05). The number of down- and up-regulated DE genes are shown at the top of each volcano plot. The y-axis denotes

-log10 p-values while the x-axis represents log2 fold change values **(B)** and confect values **(C)**. Genes that do not show a

significant effect size (FDR >0.05) have a "confect" value of NA.

**Comment 4:** Further, CAR-T cell activation and intracellular signaling differs from the native TCR- signaling due to the truncated receptor and it is unclear how relevant is the analysis.

**Answer:** We thank the reviewer for this helpful comment. In fact, the CAR-T cell manufacturing process involves activation with anti-CD3/CD28 antibody-coated beads, after which they become receptive to transduction with a gamma-retroviral vector that encodes the anti-CD19 CAR gene. After the CAR T cells have been cultured and expanded, the beads are removed (see Figure 5 below taken from Melenhorst et al. [12]). Thus, we carefully selected CAR T cells infusion bags, i.e. (CAR) T cells cultured and expanded with anti-CD3/anti-CD28 (before CAR T cells were given to the patient), as a suitable use case.

**Adjustments in the manuscript:** To better justify selection of the use case, we added in section “Discussion” the following paragraph: “Because CAR T cells are also cultured and expanded with anti- CD3/anti-CD28 beads during manufacturing [12], we aimed to analyze the activation states of the infusion product after manufacturing before the CAR T cells were infused into patients.”

**Figure. 5:** Xu J, Melenhorst JJ, Fraietta JA. Toward precision manufacturing of immunogene T-cell therapies. Cytotherapy.

2018 May;20(5):623-638. doi: 10.1016/j.jcyt.2017.12.007 . Epub 2018 Apr 10. PMID: 29653875.

**Comment 5:** Finally, there are several methodological issues that need to be addressed (see specific comments).

## Specific comments

1. The authors used RNA-seq data and microarray data, which have different dynamic ranges, and hence, the integration of both datasets is not straightforward. How was this issue solved?

**Answer:** We agree with the reviewer that the integration of RNA-Seq and microarray data is not straightforward. Therefore, we did not directly integrate microarrays and sequencing data. We analyzed each T-cell population separately and each population was either measured by microarrays or by RNA- Seq. The main analysis steps are described below (see also Supplemental section 2 of the manuscript).

- Annotation: For all array data, we used an updated probe set annotation chip definition file based on the same GENCODE annotation reference used for quantifying the RNA-Seq data. This step ensures the transferability of gene annotation between different platforms and is justified by the fact that the original annotation of the microarray data is outdated.
- DGEA (differential gene expression analysis): We used the same statistical framework for differential gene expression analysis, i.e. the R package Limma for microarrays and the Limma function voom() for RNA-Seq data. The function voom() is part of the limma R package and transfers RNA-Seq data from negative binomial models to linear models [13]. Voom() allows to fit gene expression derived from RNA-Seq by linear regression models. By following this workflow, we ensure that for both types of datasets (microarray and RNA-Seq) identical workflows are applied for (i) fitting the gene-wise statistical models and for (ii) testing the null hypothesis that coefficients do not diverge from null.
- Meta-analysis: We estimated a combined effect size for each differentially expressed (DE) gene across the T-cell populations and time points in the Discovery Set using a random effects model. We used a random effects model because we did not assume that there is one true effect size,

which is shared by all the included datasets, but rather a range of true effect sizes with additional sources or variation, such as different platforms (RNA-Seq and Microarray).

- NMF: We performed NMF for each T-cell population from the Discovery Set and Verification Sets separately. Th0, Th17 and Treg populations here consist of RNA-Seq data. Th2 and Th1 have been measured using microarrays. This means that NMF was not performed with a mixture of RNA-Seq and array samples. Subsequently, the metagenes were identified separately for each T cell population. To obtain more robust time series, we combined metagenes with similar expression patterns among CD4+ T-cell populations. This means that the metagenes had to be temporally consistent across all CD4+ T-cell populations.
- Visualization of the metagenes
  - To visualize the gene expressions in Figure 4C of the main part of the manuscript, we performed cross-platform normalization using feature-specific quantile normalization (FSQN). This normalization strategy was performed only for the mentioned figure.
  - Metagene landscape: In Figure 6A below (also Figure 4A in the main part), we mixed RNA- Seq and arrays. In Figure 6B below, we have colored the samples by platform to show that the embedding is not influenced by platform type. However, this assumes that the components used for dimension reduction have the same biological meaning between the platforms (which is the case here).

**Figure 6: Discovery Set:** Left figure is from the main part of the publication (Fig. 4a). Sample in the right figure

are colored by platform. Following the methodology described previously [14, 15], we embedded all samples relative to temporally coherent metagenes using the pattern matrix. All genes that we used for NMF were embedded relative to the temporally coherent metagenes using the gene signature matrix and depicted as density map. For IL2, IL2RA, CD4, and NF-κB (NFKBID) we calculated the average metagene weights across the

CD4^+^ T-cell populations and depicted them as dots in the metagene landscape.

**Adjustments in the manuscript:** To better describe our workflow, we thouroughly checked the manuscript and added to Section 3 in Additional File 1: “[Voom() allows to fit gene expression derived from RNA-Seq by linear regression models. By following this workflow, we ensure that for both types of datasets (microarray and RNA-Seq) identical workflows are applied for (i) fitting the gene-wise statistical models and for (ii) testing the null hypothesis that coefficients do not diverge from null. ]”

2. Non-negative matrix factorization is inherently not exactly solvable. In order to demonstrate that the results are robust, an additional techniques should be applied.

**Answer:** Thank you for your careful advice. Since NMF is NP-hard, we ran the algorithm 200 times for each rank. Pattern and gene signature matrix was obtained from the factorization that achieved the lowest approximation error for the selected rank in 200 runs as suggested by Brunet et al [16]. To determine the optimal factorization rank r (number of metagenes) for each T-cell population, we performed NMF from actual and randomized data by repeating the rank value in the interval 2–10. We used consensus clustering paired with cophenetic correlation coefficients and silhouette width plots to determine the optimal factorization rank for each T-cell population (see Figure 7 below as an example

and Figure S16 in the supplement of the publication for all T-cell populations). The same strategy has been used in a variety of studies that utilized NMF for processing genomic data, including cell type classification [17–21], mutational signature identification [22, 23], oncogenic signature dissection [15,

24], and metagene analysis [16, 25, 26].

Regarding the comment that additional techniques should be applied: Several methods, including self- organizing maps (SOM), hierarchical clustering (HC), k-means or matrix factorization techniques such as independent component analysis (ICA), and non-negative matrix factorization (NMF), can be applied to transcriptomics to reduce the dimensionality of data to get smaller set of meta-features. Brunet et al. [16] compared NMF with HC and SOM and concluded that NMF was best at elucidating the nested substructure of the data. In addition, NMF appears to be more accurate and robust to the choice of input genes than HC and more stable than SOM. Regarding the biological interpretation of the extracted components, early applications of ICA and NMF outperformed more traditional statistical techniques (HC or k-means clustering) [27]. Saidi et al. [28] came to a similar conclusion, namely that applying factorization methods to microarray data from endometrial cancer leads to a better understanding of such complex and noisy measurement environments than standard clustering algorithms.

Both ICA and NMF find metagenes associated with independent sources of variation, and both methods have been shown to have comparable performance in detecting complex biological processes [21, 29]. ICA can find both over- and underexpression of genes in a single metagene, whereas NMF will find metagenes that are additive corresponding only to overexpression of genes in that condition. As a result, ICA may better model both repression and activation than NMF, but as a side effect may have a greater mixture of metagenes than NMF [29]. A disadvantage of ICA regarding the interpretation of the results is that the basis and coefficient vectors can have positive and negative values. In the context of transcriptome data, negative values complicate the interpretation of genes. Hence, we decided to restrict the factors and coefficients to a non-negative setting. The assumption is that NMF models both the additive nature of complex biological processes and parsimony, and therefore generates solutions that are biologically intuitive to interpret.

**Figure 7 (Example of Figure S16 in supplemental material):** NMF consensus-clustering. (A) Consensus matrices averaging

200 connectivity matrices and computed for factorization ranks k = 2–10 (number of metagenes). NMF was performed for each T-cell population separately. NMF computation and model selection were performed according to Brunet et al. [16] as described in the methods. Samples are hierarchically clustered by using distances derived from consensus clustering matrix entries, colored from 0 (samples are never in the same cluster) to 1 (samples are always in the same cluster). (B-C) Cophenetic correlation coefficient for the above hierarchically clustered matrices and average silhouette-width plots. NMF was also run with randomized data (200 iterations) with same parameters as for the actual data. Red colored dots represent

the chosen factorization rank for downstream analysis.

3. The data reported in Figure 6C is significant but not very strong given the large overlap of the expression values. The reporting and the discussion should be toned down.

**Answer:** This is true, thanks for this comment. We toned down the reporting.

**Adjustments in the manuscript:** The discussion now reads: “These observations are novel with respect to ICANS [...]. It is crucial to validate these findings by further analyses including longitudinally isolated CAR T cells from patients’ peripheral blood. Notably, the metagenes were trained using samples derived from healthy donors, and not from heavily pretreated LBCL patients [51], which is important to consider.”

4. The Methods and the Results section are not clearly separated since some methodological parts are included in the Results section. The manuscript should be rewritten to distinguish the two.

**Answer:** We thank the reviewer for this advice and carefully revised Methods and Results section. All changes are in track change mode for easy traceability of text changes.

**Adjustments in the manuscript:** For example, this paragraph was moved to the methods: For each DE gene with a combined effect size, we calculated a confident effect size or “confect” [30]. This provides an inner bound on combined effect sizes while maintaining a given FDR <0.05. In this way, we ranked each gene according to its “confect”. This is a more conservative way than ranking by raw effect size because it avoids ranking genes by highest effect size, which can have a large within-group variability (see Additional file 1: Supplemental Figure S9A).

5. The discussion on the various putative genes should shortened since the genes have not been validated using orthogonal methods.

**Answer:** Thank you for this advice. We followed this reviewer’s suggestion and checked the list of genes described in section “Discussion”. Based on the UniProtKB/Swiss-Prot DB, we removed all genes for which there was no evidence at protein level and retained only the genes with the highest annotation score.

**Adjustments in the manuscript:** We removed genes EGR3, MIR155HG, and DEPDC1B from section “Discussion”.

# Reviewer #2:

In this study, Rade et al. present a first comprehensive analysis of temporal consensus gene expression signatures across T-cell populations. The authors used non-negative matrix factorization (NMF) for their analysis. They first analyzed publicly available datasets as a discovery set and then verified their findings by performing RNA-seq. Consequently, they defined temporally resolved gene expression patterns across T-cell populations after TCT stimulation, resulting in identification of 3-5 metagenes. They also used scRNA-seq datasets from large B-cell lymphoma (LBCL) patients and examined whether expression of the metagenes they identified were correlated with immune effector cell-associated neurotoxicity syndrome (ICNAS) grades.

While the findings from this study are interesting and could improve our understanding of T-cell activation after TCR stimulation, further evidence and/or analysis that support their conclusion seem to be needed. Several specific comments are as follows:

**Comment 1:** Biological significance of profiling the "consensus" gene expression across different T-cell populations seems to be unclear.

**Answer:** We thank the reviewer for this valuable comment. The rational to establish a consensus gene expression signature instead of a specific T cell subtype activation signature was the following: Immunotherapies and ATMPs represent a great opportunity in the treatment of cancer due to their immunomodulatory effects, but also come with the challenge of predicting and ensuring their efficacy and safety, when applied in humans. Prediction of immunogenicity of these drugs is based on the generation of an adaptive immune response and can be linked to an early key event, the T-cell activation [30]. Pan-T cell activation is considered as an important endpoint of immunogenicity assays and PBMCs a typical source to address this endpoint [31–33]. However, in this currently used non- clinical in vitro assays pan T cell functionality is analyzed in cytokine release assays (ELISA, ELISpot) and proliferation (FACS based CSFE or ^3^H thymidine incorporation), which are time consuming and cost intensive assays. Here, we provide a consensus signature describing T cell activation covering signatures for cytokine production and proliferation over all functional T cell states to different time points. This enables early and fast detection of immune responses in non-clinical/pre-clinical models and would not be possible with distinct signatures for each different T cell populations.

The distribution of Th1, Th2, Th17 and Th0 in human peripheral blood differs greatly from immune conditions to immune condition (e.g. cytokine storm or allergy). Although there may be clear tendencies towards certain subpopulations for well-described inflammatory diseases, for example under allergy to Th2, even these distributions vary greatly between individuals. Therefore, we determined a consensus gene expression signature that identifies T cells upon their activation across lineage choices, independently from the underlying inflammatory disease condition (e.g. inflammation vs. hypersensitivity reaction). The signature covers T cell activation independent of cellular heterogeneity.

**Adjustments in the manuscript:** We carefully revised the section “Introduction” of the manuscript to improve description of the study rationale. All modifications are highlighted with tracking marks.

(1) If the authors could address molecular mechanisms underlying how memory T-cells respond to antigenic stimulation faster than naïve T-cells, the present manuscript would be improved and of interest to many immunologists. Are M2-M5 induced faster in Memory T-cells compared to naïve T- cells?

**Answer:** We thank the reviewer for this helpful comment. For the *Memory T-cell Verification Set*, we observed a potential time shift in the activation of TNFRSF9 and IL2. For example, IL2 (see Figure 8 below) has an expression pattern peak at 6-12h in the *Discovery Set* and *Pan T-cell Verification Set*. However, in the *Memory T-cell Verification Set*, IL2 has a peak at 2-4h. This shift might be of biological origin because memory T-cells were activated in the *Memory T-cell Verification Set*, which may lead to a faster response to the activation signal. However, we were not able to confirm this observation, because the expression peak of IL2 in the *Memory T-cell Verification Set* cannot be compared with the expression peak in *Discovery Set* or *Pan T-cell Verification Set*. The *Memory T-cell Verification* Set lacks the activation time point at 6 hours. Overall, as shown in Figure 9 below (Supplemental Figure S23A), we could identify several genes, which have the same expression profile in memory T cells (dark blue) as in all other cell populations. In addition, we provide an interactive HTML document in the Supplement. Readers interested in genes that pass the discovery set but not the memory verification set can use this HTML document to explicitly filter for these genes. Classification and observed time shifts of expression can then be analyzed per gene.

It should be noted, that only two analyzed datasets (Schmidt et al. [5] and Ullah et al. [6]) explicitly mentioned that the isolated CD4+ T-cells were naïve T-cells. Thus, we believe that a large part of the analyzed data from the *Discovery Set* consists of pan-CD4 T-cells also comprising memory T-cells. For this reason, a direct comparison of potential time shifts of the metagenes between naïve and memory T-cells is unfortunately not possible. Nevertheless, the HTML document enables to elaborate for each gene of interest if its expression profile differs between pan T-cell activation and memory T cell activation.

Lastly, a month after we submitted our study, Soskic et al. [34] published a single-cell data in which naive and memory T-cells were activated for 16 h, 40 h, and 5 days. This study could serve as a source to elaborate if M2-M5 would be induced faster in memory T cells than in naïve T cells. However, in this study, the authors did not develop signatures representing differences in gene expression for activated naive and memory T-cells over time. Further, the chosen time points (16h, 40h and 5 days) are not comparable to early time points covered in our study. Thus, we were not able to identify publicly available data sets that are eligible to answer the reviewer’s questions. However, in future studies that will be conducted in our labs, we will address your highly valuable comment.

**Adjustments in the manuscript:** We revised section “Discussion” according to this comment, it now includes the following paragraph: “Although it would be of interest to address the molecular mechanisms responsible for memory T-cells responding faster to stimulation than naïve T-cells, only two data sets analyzed explicitly mentioned that the isolated CD4^+^-T cells were naïve T-cells [5, 6]. Since the other expression data sets are Pan CD4^+^ T-cells, a direct comparison of possible metagene time shifts between naive and memory T cells is not feasible.”

**Figure 8**: Additional File 2: Example of IL2 expression for each T cell population.

(2) Why don't the authors show any result from the Memory T-cell Verification Set in main figures? Alternatively, they can sort memory T-cells from some healthy donors and perform RNA-seq to address the point raised in (1).

**Answer:** We thank the reviewer for this question. Indeed, we describe the course of the metagenes in the *Memory T-cell Verification Set* in Supplemental Figure S23 (see Figure 9 below). We refrained from providing this in one of the main figures, because it would make the main figures overloaded. Furthermore, since our goal was to develop signatures that had the same expression profile over time regardless of cell type, this plot would be redundant to main Figure 4C.

With using the *Memory T-cell verification set* as an independent verification cohort, we were able to identify several genes that follow the same expression profile over time in memory T cells (dark blue) as in the other cell populations (see Figure 9 below). An additional RNA-Seq experiment would not increase the power, as the memory T-cell verification set already consists of up to 24 biological replicates per activation time point (Figure 10 below). We provide an interactive HTML document in the Supplement with which the user can filter genes that pass the discovery set but not the memory verification set. Thus, observed time shifts of expression can be analyzed per gene of interest (see our answer to the comment (1) above).

**Figure 9**: Copied from Supplementary Figure S23A: We grouped the consensus expression profiles over the course by genes

associated with identity and shared metagenes. Each boxplot represents one CD4+ T-cell population from the Discovery Set and Memory T-cell Verification Set. The y-axis depicts standardized median expression of genes from samples with identical analysis time points. The number in parentheses represents the number of genes for the corresponding metagene.

**Figure 10**: Copied from Supplemental Figure S1

**Comment 2:** Relationship between Figure 2A and B is unclear.

(1) For examples, among 5 populations, the authors identified 3641 DEGs after 24 hours of activation as described on line 337. They also identified about 3000 DEGs at each time point according to Figure 2A. Based on Figure 2A, they identified approximately 3000+3641+3500+3000 = 13141 DEGs if there are no overlap. As shown in Figure 2B, they identified 4808+26+55+67+14+7920 = 12890 DEGs. How do these two numbers (13141 and 12890) link?

**Answer:** We thank the reviewer for pointing us to the issue that Figure 2 of the manuscript was not well explained. For better understanding, the numbers for Figure 2A are shown below (see also Figure 11 below). The exact numbers are 2999 + 3641 + 3581 + 2669 = 12890. The sum of the red numbers (i.e. number of DE genes in five T cell populations) in Figure 11A (Figure 2A in the main part of the manuscript) is the same as the sum of the red numbers in Figure 11B. However, the sum of the green, blue, grey and black numbers in Figure 11A and B are not identical. This is because Figure 11A shows an inverse cumulative distribution of significantly differentially expressed genes (i.e. numbers in Figure 11A correspond to number of DE genes in at least 4, 3, 2, or 1 T cell population, respectively). This means, for example, that after 24 hours of activation we found 5,132 significant genes in at least four T-cell populations (red circle). Figure 11B (Figure 2B in the main part), on the other hand, shows the number of T-cell populations in which genes were identified as DE (y-axis) and the number of genes with consistent fold changes across T-cell populations (x-axis).

With respect to your comment: “*Based on Figure 2A, they identified approximately 3000 + 3641 + 3500*

*+ 3000 = 13141 DEGs if there are no overlap*”: there is an overlap between differentially expressed

genes (DEGs). For example, genes that are significant in all five T-cell populations after 12 hours may also be significant after 24 hours (Figure 11A below, red curve).

**Figure 11:** With the exception of the numbers in A), this figure is the same as Figure 2 from the main section.

**Adjustments in the manuscript:** In order to better explain Figure 2, we revised legend of Figure 2 in the main part of the manuscript and added the values of the cumulative sums to Figure 2A (see Figure 12 below). We have also plotted the values of the cumulative sums and recolored the curves to show that A) has a different meaning than B) and C).

**Figure 12:** Revised Figure 2 from the main part of the manuscript.

In addition, on lines 673-674, the authors argue that 7920 genes were upregulated in all 5 population "at each contrast" (e.g. 12h vs. 0h). The meaning of "at each contrast" is unclear for this reviewer.

**Answer:** Thank you for pointing this out. That is indeed confusing. For example, consider the number 11,020 in Figure 11B above (highlighted in red). Here, for example, gene 1 (out of 11,020 genes) could be significantly upregulated after 12 hours of activation in both T cell population A and B. However, if gene 1 were significantly upregulated in T cell population A after 6 hours of activation and significantly upregulated in population B after 12 hours, this gene would not appear in the figure. The meaning of contrast (e.g. 12h vs. 0h) is as follows: For example, in differential gene expression analysis, we formed

two groups for population A: samples 12 h after activation and samples before activation (0 h). We did the same for all other T-cell populations.

**Adjustments in the manuscript:** We have simplified the caption as follows: “… .These DE genes were also upregulated in all 5 T-cell populations at the same time point of activation. …”

(2) For another example, as show in black in Figure 2A, they identified >10000 DEGs at each time point except at 1h, resulting in identification of at least 70000 DEGs. Based on Figure 2B, they identified 17867+14097= 31964 DEGs. How do these two numbers (i.e. 70000 and 31964) link? Is there any overlap?

**Answer:** The sum of all numbers from the black line is 113,002. If we add up all the numbers in Figure 2B) we also get 113,002. The reason for this is that Figure 2A) represents an inverse cumulative distribution. One further example: the sum of all number from the green line is 26,523. If we add up all green and red number from B), we also get 26,523.

(3) This reviewer assumes that union of DEGs at each time point is equal to summation of gene number in each color. Venn diagram would be helpful for understanding the relationship between Figure 2A and B.

**Answer:** Thanks you for carefully checking Figure 2 in the manuscript. Since, Figures 2A and B have different objectives, we did not generate a Venn diagram but revised content, caption and legend of Figure 2.

**Adjustments in the manuscript:** We revised content, caption and legend of Figure 2 in the manuscript.

**Minor points**

1. In Figure 6B, "standardized average expression" should be added beside the color key.

**Adjustments in the manuscript:** Thank you very much for this hint. We have now used a uniform color key for all 3 dimension reductions and added the title "standardized average expression" (Figure 13 below red circle).

Figure 13: Revised Figure 6 from the main part of the manuscript.

2. In Figure 6C, it is supposed that T-cells are more active in patients with higher grade of ICANS than those with lower grade. However, the authors observed an opposite result: T-cells showed decreased

expression of M5, M3, and M3/M5 in patients with higher grade of ICANS. The authors should discuss this matter.

**Answer:** Thank you for raising this point that seems counterintuitive at first sight. A positive correlation between proliferation, activation as well as peak expansion and severity/duration of ICANS/CRS has been observed in vivo, especially for CAR T-cells directed against CD19 [35]. An increase of pro- inflammatory cytokines in the serum of the respective patients precedes the respective activation of CAR T-cells.

As the reviewer noted, metagenes M3, M5 and M3/M5, were enriched in CAR T cell infusion products from patients with low-grade ICANS compared to patients with high-grade ICANS. This observation seems contrary to what one would expect. However, in our current analysis, we studied infusion products instead of in vivo activated CAR T-cells following infusion. In the analyzed dataset from Deng et al., ICANS grade 3 and 4 occurred in median 4 days after infusion. This underlines that activation and proliferation as well as peak expansion of CAR T-cells that were responsible for the respective side effects most likely to have occurred after infusion and subsequent activation of CAR T-cells through antigen exposure on target malignant B-cells. Therefore, the infusion of already activated and proliferative CAR T-cells was not linked to the occurrence of higher grade ICANS.

Future studies are necessary that will analyze longitudinally isolated CAR T-cells to confirm our hypothesis that especially expansion and activation of CAR T-cells in vivo after infusion are mainly responsible for ICANS, while the application of an infusion product that contains already proliferating CAR T cells prior to antigen exposure protects from higher grade ICANS. Currently, no dataset with a reasonable number of biological replicates of time-resolved transcriptome studies of cells derived from peripheral blood and documentation of ICANS is publicly available that could be used to address this.

**Adjustments in the manuscript:** We carefully revised the section “Discussion” to improve interpretation of results of Figure 6. All modifications are highlighted with tracking marks.

3. On line 542, a term "early-response metagene (M1)" seems to be strange because expression of M1 decreases rapidly after TCR stimulation. "early-response" is more suitable for M2, which increases rapidly after TCR stimulation. Please consider renaming of M1.

**Answer:** Thank you for pointing this out. We renamed M1 to “sustained repressed”.

References

1. Hess K, Yang Y, Golech S, Sharov A, Becker KG, Weng N-P. Kinetic assessment of general gene

expression changes during human naive CD4+ T cell activation. Int. Immunol. 2004;16:1711–21.

2. Elo LL, Järvenpää H, Tuomela S, Raghav S, Ahlfors H, Laurila K, et al. Genome-wide Profiling of Interleukin-4 and STAT6 Transcription Factor Regulation of Human Th2 Cell Programming.

Immunity. 2010;32:852–62.

3. Aijö T, Edelman SM, Lönnberg T, Larjo A, Kallionpää H, Tuomela S, et al. An integrative computational systems biology approach identifies differentially regulated dynamic

transcriptome signatures which drive the initiation of human T helper cell differentiation. BMC Genomics. 2012;13:572.

4. Tuomela S, Rautio S, Ahlfors H, Öling V, Salo V, Ullah U, et al. Comparative analysis of human and mouse transcriptomes of Th17 cell priming. Oncotarget. 2016;7:13416–28.
5. Schmidt A, Marabita F, Kiani NA, Gross CC, Johansson HJ, Éliás S, et al. Time-resolved

transcriptome and proteome landscape of human regulatory T cell (Treg) differentiation reveals novel regulators of FOXP3. BMC Biol. 2018;16:47.

6. Ubaid Ullah N, Andrabi SBA, Tripathi SK, Dirasantha O, Kanduri K, Rautio S, et al. Transcriptional Repressor HIC1 Contributes to Suppressive Function of Human Induced Regulatory T Cells. Cell Rep. 2018;22:2094–106.
7. Gutierrez-Arcelus M, Baglaenko Y, Arora J, Hannes S, Luo Y, Amariuta T, et al. Allele-specific

expression changes dynamically during T cell activation in HLA and other autoimmune loci. Nat. Genet. 2020;52:247–53.

8. Soon MSF, Lee HJ, Engel JA, Straube J, Thomas BS, Pernold CPS, et al. Transcriptome dynamics of CD4+ T cells during malaria maps gradual transit from effector to memory. Nat. Immunol. 2020;21:1597–610.
9. Richard AC, Lun ATL, Lau WWY, Göttgens B, Marioni JC, Griffiths GM. T cell cytolytic capacity is independent of initial stimulation strength. Nat. Immunol. 2018;19:849–58.
10. Lönnberg T, Svensson V, James KR, Fernandez-Ruiz D, Sebina I, Montandon R, et al. Single-cell RNA-seq and computational analysis using temporal mixture modelling resolves Th1/Tfh fate bifurcation in malaria. Sci Immunol. 2017;2.
11. Chapman NM, Chi H. Hallmarks of T-cell Exit from Quiescence. Cancer Immunol Res. 2018;6:502– 8.
12. Xu J, Melenhorst JJ, Fraietta JA. Toward precision manufacturing of immunogene T-cell therapies. Cytotherapy. 2018;20:623–38. doi:10.1016/j.jcyt.2017.12.007.
13. Law CW, Chen Y, Shi W, Smyth GK. voom: precision weights unlock linear model analysis tools for RNA-seq read counts. Genome Biol. 2014;15:R29.
14. Wu Y, Tamayo P, Zhang K. Visualizing and Interpreting Single-Cell Gene Expression Datasets with Similarity Weighted Nonnegative Embedding. Cell Syst. 2018;7:656-666.e4.
15. Kim JW, Abudayyeh OO, Yeerna H, Yeang C-H, Stewart M, Jenkins RW, et al. Decomposing

Oncogenic Transcriptional Signatures to Generate Maps of Divergent Cellular States. Cell Syst. 2017;5:105-118.e9.

16. Brunet J-P, Tamayo P, Golub TR, Mesirov JP. Metagenes and molecular pattern discovery using matrix factorization. Proc. Natl. Acad. Sci. U. S. A. 2004;101:4164–9.
17. Lin B-B, Lei H-Q, Xiong H-Y, Fu X, Shi F, Yang X-W, et al. MicroRNA-regulated transcriptome

analysis identifies four major subtypes with prognostic and therapeutic implications in prostate cancer. Computational and structural biotechnology journal. 2021;19:4941–53.

doi:10.1016/j.csbj.2021.08.046.

18. Meng J, Zhou Y, Lu X, Bian Z, Chen Y, Zhou J, et al. Immune response drives outcomes in prostate cancer: implications for immunotherapy. Molecular oncology. 2021;15:1358–75.

doi:10.1002/1878-0261.12887.

19. You S, Knudsen BS, Erho N, Alshalalfa M, Takhar M, Al-Deen Ashab H, et al. Integrated

Classification of Prostate Cancer Reveals a Novel Luminal Subtype with Poor Outcome. Cancer research. 2016;76:4948–58. doi:10.1158/0008-5472.CAN-16-0902.

20. Shao C, Höfer T. Robust classification of single-cell transcriptome data by nonnegative matrix factorization. Bioinformatics. 2017;33:235–42. doi:10.1093/bioinformatics/btw607.
21. Kotliar D, Veres A, Nagy MA, Tabrizi S, Hodis E, Melton DA, Sabeti PC. Identifying gene expression programs of cell-type identity and cellular activity with single-cell RNA-Seq. eLife Sciences

Publications, Ltd. 07.08.2019.

22. Alexandrov LB, Nik-Zainal S, Wedge DC, Campbell PJ, Stratton MR. Deciphering signatures of mutational processes operative in human cancer. Cell reports. 2013;3:246–59.

doi:10.1016/j.celrep.2012.12.008.

23. Pal S, Bi Y, Macyszyn L, Showe LC, O'Rourke DM, Davuluri RV. Isoform-level gene signature

improves prognostic stratification and accurately classifies glioblastoma subtypes. Nucleic acids research. 2014;42:e64. doi:10.1093/nar/gku121.

24. Reilly B, Tanaka TN, Diep D, Yeerna H, Tamayo P, Zhang K, Bejar R. DNA methylation identifies genetically and prognostically distinct subtypes of myelodysplastic syndromes. Blood advances. 2019;3:2845–58. doi:10.1182/bloodadvances.2019000192.
25. Moffitt RA, Marayati R, Flate EL, Volmar KE, Loeza SGH, Hoadley KA, et al. Virtual microdissection identifies distinct tumor- and stroma-specific subtypes of pancreatic ductal adenocarcinoma. Nature genetics. 2015;47:1168–78. doi:10.1038/ng.3398.
26. Li YE, Xiao M, Shi B, Yang Y-CT, Wang D, Wang F, et al. Identification of high-confidence RNA regulatory elements by combinatorial classification of RNA-protein binding sites. Genome biology. 2017;18:169. doi:10.1186/s13059-017-1298-8.
27. Liebermeister W. Linear modes of gene expression determined by independent component analysis. Bioinformatics. 2002;18:51–60. doi:10.1093/bioinformatics/18.1.51.
28. Saidi SA, Holland CM, Kreil DP, MacKay DJC, Charnock-Jones DS, Print CG, Smith SK. Independent component analysis of microarray data in the study of endometrial cancer. Oncogene. 2004;23:6677–83. doi:10.1038/sj.onc.1207562.
29. Stein-O'Brien GL, Arora R, Culhane AC, Favorov AV, Garmire LX, Greene CS, et al. Enter the Matrix: Factorization Uncovers Knowledge from Omics. Trends in genetics : TIG. 2018;34:790– 805. doi:10.1016/j.tig.2018.07.003.
30. Rosenberg AS, Sauna ZE. Immunogenicity assessment during the development of protein

therapeutics. Journal of Pharmacy and Pharmacology. 2018;70:584–94. doi:10.1111/jphp.12810.

31. Duke BR, Mitra-Kaushik S. Current In Vitro Assays for Prediction of T Cell Mediated

Immunogenicity of Biotherapeutics and Manufacturing Impurities. J Pharm Innov. 2020;15:202–

18. doi:10.1007/s12247-019-09412-5.

32. Singh SK. Impact of product-related factors on immunogenicity of biotherapeutics. Journal of Pharmaceutical Sciences. 2011;100:354–87. doi:10.1002/jps.22276.
33. Bray-French K, Hartman K, Steiner G, Marban-Doran C, Bessa J, Campbell N, et al. Managing the Impact of Immunogenicity in an Era of Immunotherapy: From Bench to Bedside. Journal of

Pharmaceutical Sciences. 2021;110:2575–84. doi:10.1016/j.xphs.2021.03.027.

34. Soskic B, Cano-Gamez E, Smyth DJ, Ambridge K, Ke Z, Matte JC, et al. Immune disease risk variants regulate gene expression dynamics during CD4+ T cell activation. Nat Genet. 2022;54:817–26. doi:10.1038/s41588-022-01066-3.
35. Morris EC, Neelapu SS, Giavridis T, Sadelain M. Cytokine release syndrome and associated

neurotoxicity in cancer immunotherapy. Nat Rev Immunol. 2022;22:85–96. doi:10.1038/s41577- 021-00547-6.

**Second round of review**

**Reviewer 1**

The authors addressed all issues raised in the previous review satisfactorily.

**Reviewer 2**

The authors have adequately addressed the comments made by this reviewer and revised the manuscript accordingly.
Therefore, I have no further comments.
